# Genetic Modification of *KNAT7* Transcription Factor Expression Enhances Saccharification and Reduces Recalcitrance of Woody Biomass in Poplars

**DOI:** 10.3389/fpls.2021.762067

**Published:** 2021-10-26

**Authors:** Yogesh Kumar Ahlawat, Akula Nookaraju, Anne E. Harman-Ware, Crissa Doeppke, Ajaya K. Biswal, Chandrashekhar P. Joshi

**Affiliations:** ^1^Department of Biological Sciences, Michigan Technological University, Houghton, MI, United States; ^2^Department of Horticultural Sciences, University of Florida, Gainesville, FL, United States; ^3^Kaveri Seed Company Limited, Secunderabad, India; ^4^Renewable Resources and Enabling Sciences Center, National Renewable Energy Laboratory, Golden, CO, United States; ^5^Department of Biochemistry and Molecular Biology, University of Georgia, Athens, GA, United States; ^6^Complex Carbohydrate Research Center, University of Georgia, Athens, GA, United States

**Keywords:** antisense, developing xylem, overexpression, saccharification, secondary cell wall biosynthesis

## Abstract

The precise role of KNAT7 transcription factors (TFs) in regulating secondary cell wall (SCW) biosynthesis in poplars has remained unknown, while our understanding of KNAT7 functions in other plants is continuously evolving. To study the impact of genetic modifications of homologous and heterologous *KNAT7* gene expression on SCW formation in transgenic poplars, we prepared poplar *KNAT7* (*PtKNAT7*) overexpression (*PtKNAT7*-OE) and antisense suppression (*PtKNAT7*-AS) vector constructs for the generation of transgenic poplar lines *via Agrobacterium*-mediated transformation. Since the overexpression of homologous genes can sometimes result in co-suppression, we also overexpressed *Arabidopsis KNAT7* (*AtKNAT7-OE*) in transgenic poplars. In all these constructs, the expression of *KNAT7* transgenes was driven by developing xylem (DX)-specific promoter, DX15. Compared to wild-type (WT) controls, many SCW biosynthesis genes downstream of KNAT7 were highly expressed in poplar *PtKNAT7-OE* and *AtKNAT7-OE* lines. Yet, no significant increase in lignin content of woody biomass of these transgenic lines was observed. *PtKNAT7-AS* lines, however, showed reduced expression of many SCW biosynthesis genes downstream of *KNAT7* accompanied by a reduction in lignin content of wood compared to WT controls. Syringyl to Guaiacyl lignin (S/G) ratios were significantly increased in all three *KNAT7* knockdown and overexpression transgenic lines than WT controls. These transgenic lines were essentially indistinguishable from WT controls in terms of their growth phenotype. Saccharification efficiency of woody biomass was significantly increased in all transgenic lines than WT controls. Overall, our results demonstrated that developing xylem-specific alteration of *KNAT7* expression affects the expression of SCW biosynthesis genes, impacting at least the lignification process and improving saccharification efficiency, hence providing one of the powerful tools for improving bioethanol production from woody biomass of bioenergy crops and trees.

## Introduction

Plant cell walls serve as significant sinks for the irreversible sequestering of fixed atmospheric carbon (Pauly and Keegstra, 2008). Lignocellulosic biomass from secondary cell walls (SCW) is one of the most promising bioenergy feedstocks for producing second-generation bioethanol (Demura and Ye, 2010; Nookaraju et al., 2013; Zhong et al., 2019). The unraveling of the poplar genome sequence by Tuskan et al. (2006) has opened up many new avenues into the production of transgenic poplars with altered expression of cell wall genes for enhanced saccharification and biomass growth assisting in an efficient bioconversion of cell wall biomass to bioethanol (Sahoo and Maiti, 2014; Busov, 2018; Cho et al., 2019; Bryant et al., 2020). A battery of transcription factors (TFs) regulates SCW formation (Zhong and Ye, 2014). For example, Zhong et al. (2008) suggested that NAC TFs are the top-tier master regulators directly activating the expression of several lower-level TFs, including MYBs and KNAT7. Second-tier regulators like MYB46 also directly regulate the expression of third-tier targets like KNAT7, impacting the biosynthesis of SCW components, namely, cellulose, xylan, and lignin (Ko et al., 2012a; Rao and Dixon, 2018). Thus, KNAT7 TF expression is directly or indirectly regulated by upstream regulators, but KNAT7 also plays an essential role in SCW formation. Genetic manipulation of *KNAT7* gene expression in poplars has not yet been reported, but such knowledge will assist in using transgenic woody biomass for bioethanol production in the future.

In *Arabidopsis*, *AtKNAT7* (KNOTTED ARABIDOPSIS THALIANA) is one of the *KNOX* (KNOTTED1-like homeodomain) TF gene family members, which is highly conserved across the angiosperms (Rao and Dixon, 2018; Ma et al., 2019). There are at least two main classes of *KNOX* genes. Class I *KNOX* genes play an essential role in meristem function, leaf development, and hormone homeostasis (Hake et al., 2004). In contrast, the functions of Class II *KNOX* genes have primarily remained unclear until recently, although some members were reported to be involved in root development (e.g., Hay and Tsiantis, 2010). *AtKNAT7*, belonging to the *AtKNOX II* gene family, recently came into the limelight because of its paradoxical role in regulating SCW biosynthesis (Zhong et al., 2008; Li et al., 2012). It was suggested that AtKNAT7 is a negative regulator (or transcriptional repressor) of the downstream genes involved in SCW formation in *Arabidopsis* because, in the knockout mutant *Atknat7*, significant thickening in the secondary walls of interfascicular fibers was observed than the wild type plants. However, SCWs of vessels of *Atknat7* mutant remained thin-walled and showed irregular xylem (*irx*) or collapsed xylem phenomenon due to weak SCWs. Thus, AtKNAT7 appears to be differentially regulating SCW formation in different cells/tissues of the same plant. *AtKNAT7* was also shown to be a functional ortholog of poplar *KNAT7* (*PtKNAT7*) because *PtKNAT7* complemented the *Atknat7* mutant (Li et al., 2012). Overexpression of *AtKNAT7* in wild-type *Arabidopsis* showed thinning in the interfascicular fiber walls, and *RT-PCR* results showed upregulation of many SCW biosynthetic genes in *Atknat7* mutant (Li et al., 2012). In contrast, primary cell wall gene expression remained unaffected. Collectively, these results suggested that AtKNAT7 TF is a negative regulator of SCW formation in fiber walls (but perhaps a positive regulator of SCW synthesis in vessel walls). However, contrasting results were earlier reported by Zhong et al. (2008) in *Arabidopsis* expressing engineered dominant repression variant of AtKNAT7. Dominant repression of AtKNAT7 caused a severe reduction in secondary wall thickening in both interfascicular fibers and intrafascicular xylem fibers in inflorescence stems, suggesting that AtKNAT7 TF is a positive regulator of SCW formation in fibers. The severity of reduction in cell wall thickness correlated with the degree of repression. Similarly, Pandey et al. (2016) identified *NbKNAT7* as a positive regulator of SCW formation in tobacco by using virus-induced gene silencing (VIGS) experiments. The *NbKNAT7* overexpression lines had thicker cell walls, suggesting that KNAT7 TF is a positive regulator of the SCW biosynthesis in tobacco. Also, the saccharification from SCW biomass of the VIGS *NbKNAT7 lines* was 40% higher than the controls. It was recently reported that KNAT7 also positively controls the xylan biosynthesis pathway by binding to the promoter of the *IRX9* gene, and expression studies showed that the transcript levels of *IRX9*, *IRX10*, and *FRA8* genes were lower in the *Atknat7* mutant (He et al., 2018). So, the data reported so far regarding the role of KNAT7 in SCW regulation appears to be confusing and contrasting. No studies have been reported in woody plant species like poplars regarding the precise role of KNAT7 during SCW formation.

The main goal of this research was to understand how genetic modifications in the expression of the *PtKNAT7* gene affect the SCW formation and study its impact on the saccharification efficiency of lignocellulosic biomass from such transgenic poplars. Here, we investigated the role of *PtKNAT7* in poplars by creating overexpression and antisense suppression lines of transgenic poplar. In addition, we present results of heterologous overexpression of *AtKNAT7* in transgenic poplars to examine the impact of such genetic manipulations on SCW formation and saccharification properties in transgenic poplars. These experiments were set to circumvent the co-suppression that commonly occurs during the overexpression of native genes due to high sequence homology.

## Materials and Methods

### Vector Construction and Plant Transformation

The *PtKNAT7* gene overexpression (sense) and suppression (antisense) vector constructs were designated as *PtKNAT7-OE* and *PtKNAT7-AS*. The full-length *KNAT7* cDNA from poplar stems was amplified using gene-specific primers (see Supplementary Table 1) with integrated *Xba*I and *Sac*I Restriction Enzyme sites and inserted into the DX15pBI101 vector by replacing the GUS gene (Kindly provided by Dr. K-H Han, Michigan State University). The *PtKNAT7* insert was flipped over in the opposite direction for the antisense construct from the sense constructs. For the second part of this study, we overexpressed *AtKNAT7* in poplars using the same DX15 promoter and designated this construct as *AtKNAT7*-OE. For this vector construct, *AtKNAT7* cDNA was amplified using gene-specific primers and ligated into *Xba*I/*Sac*I digested DX15pBI101 vector to replace the GUS gene. All the three constructs, *PtKNAT7*-OE, *PtKNAT7*-AS, and *AtKNAT7*-OE, were used for the transformation of hybrid poplar (*Populus tremula* × *Populus alba* clone 717-1 B4) explants using *Agrobacterium* C58 strain by leaf disc infiltration method (Liu et al., 2012).

### RNA Extraction and Gene Expression Studies

Total RNA was isolated from Stem Differentiating Xylem (SDX) scraped from 20 to 50 aerial internodes of hybrid poplar plants using the TRIZOL (Ambion, Life Technologies) method as described earlier (Liu et al., 2012). Extracted RNA was treated with DNase (Turbo DNA free, Thermo-fisher). First-strand cDNA synthesis was performed using 1 μg of total RNA using reverse transcription kit (Applied Bio-systems). qRT-PCR was performed using PowerUp SYBR green master mix (Applied Biosystems). Real-time primers were designed for poplar *KNAT7* and Arabidopsis *KNAT7* using Integrated DNA Technologies (IDT) software. The total reaction was 12 μl containing 6 μl of SYBR green, 1 μl of each primer (1 μM), 1 μl of cDNA template, and 3 μl of RNase-free water. The reaction for each gene was performed in triplicates with thermocycler conditions as follows: 95°C for 10 min followed by 45 cycles for 95°C for 30 s, and 60°C for 30 s. Relative gene expression was calculated by the ΔCT method. Actin7 was used as an internal control (Zhang et al., 2010).

### Microscopic Studies

For histology, sections were cut at the seventh internode from the apex of poplar plants (with about 50 internodes above the soil) and preserved in ice-cold FAA (37% formaldehyde, glacial acetic acid, and 95% ethanol). The internodes were infiltrated under a vacuum for about 10–15 min and kept overnight at 4°C. Dehydration and embedding in wax were done for fixing the sections on slides. Dewaxing was performed using two washes of xylene and decreasing concentrations of ethanol followed by washing with water. Autofluorescence was observed from dewaxed sections using fluorescent light imaging.

### Growth Measurements

The growth characteristics of the greenhouse-grown plants were measured every week. All the independent lines for each gene construct were grown under identical conditions in the greenhouse as described by Liu et al. (2012). Three parameters, namely, stem height, stem thickness, and the number of leaves were measured for one-month-old plants from week 1 until the day of harvest in week 9 in the greenhouse.

### Lignin Analysis Using Pyrolysis-Molecular Mass Beam Spectrometry

After scraping the developing xylem from transgenic plants, the wood was dried at room temperature for 2 weeks and then milled through a 20-gage mesh filter. One gram of wood powder was stored in falcon tubes, destarched with amylase and ethanol extracted (described in the following section), and 4 mg of destarched-extracted material was used for lignin analysis. Three samples from each gene construct were sent to National Renewable Energy Laboratory (NREL) for analysis by Pyrolysis-Molecular Mass Beam Spectrometry (Py-MBMS) to estimate lignin content and for S/G ratio measurement (Decker et al., 2018). Duplicate samples were analyzed for each line.

### Saccharification Assays

Pretreatment and enzymatic hydrolysis of biomass was carried out as described previously with some modifications (Biswal et al., 2015, 2018). Before the analysis, biomass was treated with alpha-amylase (0.47 U per mg biomass, Sigma Cat # A6255) in 100 mM ammonium formate (pH 5.0) buffer at 25°C for 48 h to remove starch, followed by three water and two acetone washes. Then biomass samples were kept under the hood for 72 h for drying. This was followed by an ethanol Soxhlet extraction for an additional 24 h to remove extractives. After drying overnight, pretreatment was carried out with 5 mg of dry biomass at 180°C for 17.5 min. About 40 μl of buffer-enzyme stock, 8% CTec2 (Novozymes) in 1.0 M sodium citrate buffer, was added to the pretreated biomass. The samples were incubated at 50°C for 70 h. After 70 h of incubation, the hydrolysate was analyzed using Megazyme’s GOPOD and XDH assays as described earlier (Biswal et al., 2015, 2018).

## Results

### Gene Expression Studies in *KNAT7* Transgenic Poplar Lines

**FIGURE 1:**
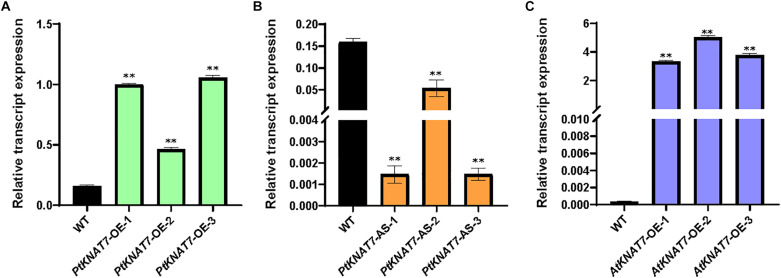
Relative transcript expression was estimated through quantitative RT-PCR using three independent transgenic lines in each of the poplar gene constructs: *PtKNAT7-OE* **(A)**, *PtKNAT7-AS* **(B)**, and *AtKNAT7-OE* **(C)**. Sampling was done in triplicate of each sample of the transgenic line.

**FIGURE 2:**
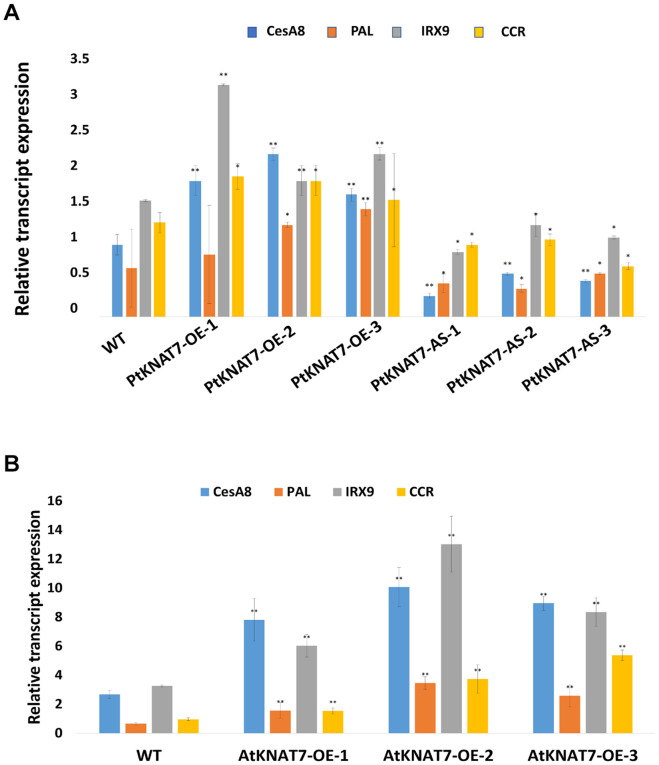
Effect of *KNAT7* transgene expression on four selected SCW biosynthetic genes in poplars, namely, *CesA8, PAL, IRX9*, and *CCR*. **(A)** SCW gene expressions patterns in *PtKNAT7-OE* and *PtKNAT7-AS* transgenic lines. **(B)** SCW gene expressions patterns in *AtKNAT7-OE* transgenic lines. Bars represent the average of three replicates of three transgenic lines. All differences were significant when compared to WT controls.

The relative expression of the *KNAT7* gene in wild-type control (WT) and transgenic *KNAT7* poplars were quantified using qRT-PCR. The *PtKNAT7* gene transcripts were significantly increased in *PtKNAT7*-OE lines compared to the WT plants (Figure 1A), whereas significant suppression of *PtKNAT7* transcript formation was observed in *PtKNAT7-AS* transgenic trees (Figure 1B). Figure 1C shows the substantial upregulation in *AtKNAT7* transcripts in *AtKNAT7-OE* transgenic poplar lines. We used the same *PtKNAT7-OE, PtKNAT7-AS*, and *AtKNAT7-OE* lines for further analyses. We selected four representative poplar SCW genes, namely *CesA8, IRX9, PAL*, and *CCR* for the expression studies in these transgenic poplar lines. In the case of *PtKNAT7-OE* lines, all four SCW genes were upregulated and the same genes were significantly downregulated in *PtKNAT7-AS* lines (Figure 2A). We also observed that the same four SCW biosynthesis genes were also upregulated in the *AtKNAT7-O*E lines of transgenic poplar lines (Figure 2B). These results suggest that KNAT7 TF is involved in the expression of downstream SCW genes and transgenic plants show expected altered expression of SCW genes.

### Growth Measurements of Transgenic *KNAT7* Poplar Plants

**FIGURE 3:**
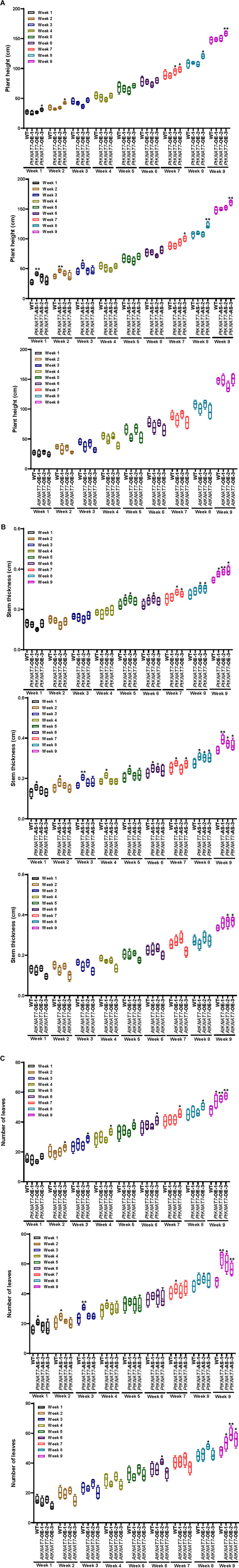
Phenotypic measurements were performed every week in WT control and transgenic poplars until the day of harvest in the 9th week. **(A)** Plant Height, **(B)** stem thickness, and **(C)** the number of leaves in *PtKNAT7*-OE, *PtKNAT7*-AS, and AtKNAT7-OE were measured for 9 weeks until the day of harvest. The data is represented as a means ± SD (*n* = 3).

Once the homologous or heterologous *KNAT7* gene overexpression or silencing was confirmed in a variety of transgenic poplar *KNAT7* lines using gene expression studies, the same three independent transgenic lines for each construct were chosen for measuring the growth parameters. The plant height, stem thickness, and the number of leaves measurements were recorded every week until the day of harvest in about 9 weeks (Figures 3A–C). The height of most of the three transgenic lines was not significantly greater than WT plants, except for the *PtKNAT7-OE-3* line, and *PtKNAT7-AS-3* line (Figure 3A) by week 9 but eight of the nine transgenic lines examined had significantly increased stem diameter (Figure 3B) than the WT plants by week 9. In terms of the number of leaves in all three types of transgenic lines, there was a significant increase as compared to WT plants by week 9 (Figure 3C). Thus, growth phenotypes of most of the transgenic *KNAT7* lines examined were either similar or a little better than WT lines by the time of harvest (Supplementary Figure 1).

### Transgenic *KNAT7* Poplar Lines Show Altered Xylem Growth Phenotype

**FIGURE 4:**
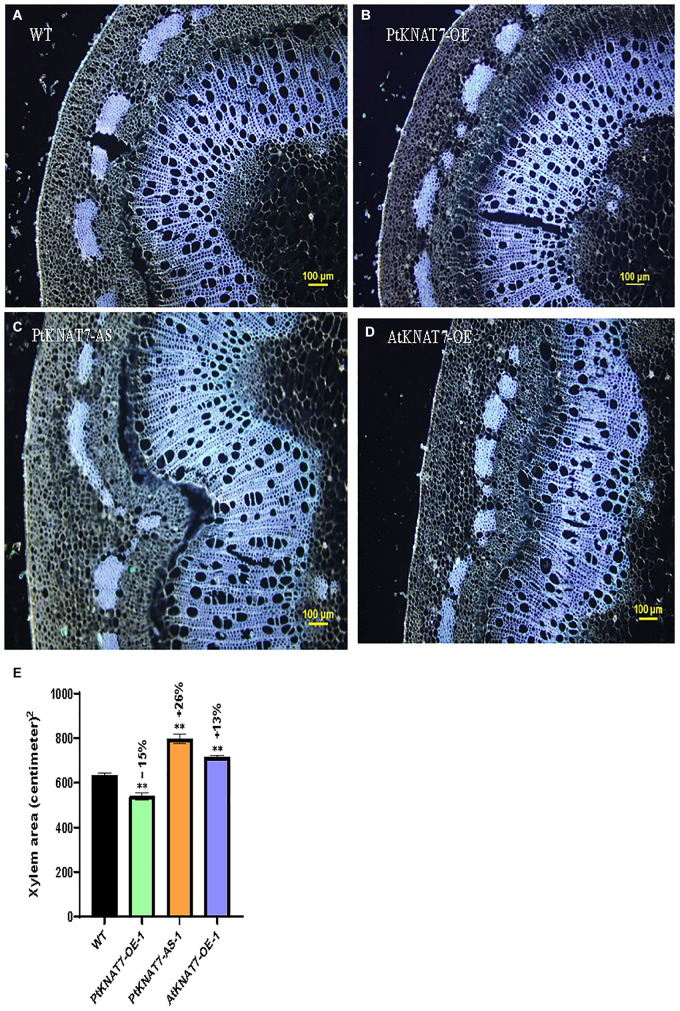
Autofluorescence images of cross-sections and measurements of xylem area. Tissue sections for histological studies were performed at the 7th internode from the top. Cross-section of wild type **(A)** compared from *PtKNAT7*-OE **(B)**, *PtKNAT7*-AS **(C)**, *AtKNAT7*-OE **(D)**, Scale bars-100 micrometers. **(E)** Xylem area measurements in WT and three *KNAT7* transgenic lines in poplars. Significant differences were observed as compared to WT controls. Measurements were calculated from the xylem area observed at three separate points and averaged (*n* = 3).

Pandey et al. (2016) reported that genetic modifications of the *KNAT7* gene expression in tobacco showed the altered amount of xylem occupying stem sections. VIGS and *RNAi*-suppression lines for the *NbKNAT7* gene showed about 50% increase in xylem area with reduced xylem fiber wall thickness whereas *NbKNAT7* overexpression lines showed increased xylem cell wall thickness but did not show such increase in xylem area. Here, we used autofluorescence of lignin in the dewaxed stem sections from the top 7th internode to estimate the xylem area in transgenic *KNAT7* poplars compared to WT plants (Figures 4A–E). In the case *of PtKNAT7*-OE overexpression lines (Figures 4B,E), we observed a 15% decrease in the xylem area as compared to controls (Figures 4A,E) whereas a 26% increase in the xylem area was observed in the *PtKNAT7*-AS lines (Figures 4C,E) over the WT control plants (Figures 4A,E). About a 13% increase in the xylem area was observed in *AtKNAT7*-OE sections (Figures 4D,E) over control. Qualitatively, a slight increase in the lignin autofluorescence signal was also observed in the *KNAT7* overexpression lines of poplars as compared to the stem sections of WT control plants (Figures 4A,B). However, no changes in autofluorescence levels were observed in the case of the *AtKNAT7*-OE lines than controls (Figure 4D). There were no obvious differences in the fiber or vessel wall thickness in any of the *KNAT7* transgenic lines and these observations are similar to a previous report by Zhong et al. (2008).

### Changes in *KNAT7* Gene Expression Leads to Altered Lignin Content and Constitution in Some Transgenic Lines

**FIGURE 5:**
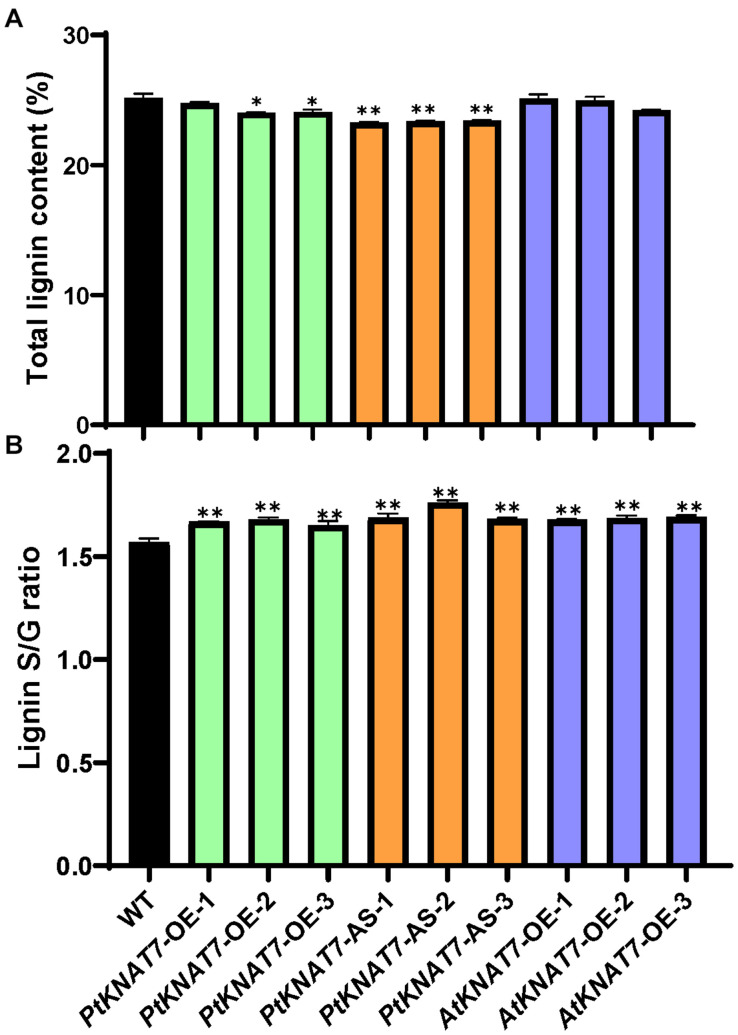
Lignin content and S/G lignin ratio measurements in poplar transgenic lines: **(A)** Lignin contents in three *PtKNAT7-OE, PtKNAT7-AS*, and *AtKNAT7-OE* transgenic lines in poplar. **(B)** S/G lignin ratio measurements in three *PtKNAT7-OE, PtKNAT7-AS*, and *AtKNAT7-OE* transgenic lines in poplar. Data analyses were performed in duplicate for each sample. Significant observations are marked by * when compared to WT controls (*P*-value < 0.05).

Lignin analysis was performed using py-MBMS. No significant changes in lignin content were observed for *PtKNAT7-OE* lines, but there was a significant decrease of about 6% lignin in the case of *PtKNAT7-AS* lines as compared to the controls (Figure 5A). *AtKNAT7-OE* expression lines also did not show any observable changes in lignin content. However, the S/G lignin ratios were significantly increased in all three *KNAT7* transgenic lines (Figure 5B). Overall, a 5–7% increase in *PtKNAT7-OE*, 8–12% increase in *PtKNAT7-AS* lines, and 7% increase in *AtKNAT7-OE* lines were observed for the S/G lignin ratios as compared to controls. A change in the S/G lignin ratio could bring about structural and other property changes in the cell walls. Therefore, we further explored how these changes in the cell wall composition resulted in changes in the saccharification efficiency.

### Saccharification Efficiency Was Significantly Improved in the Genetically Manipulated *KNAT7* Transgenic Lines of Poplars

**FIGURE 6:**
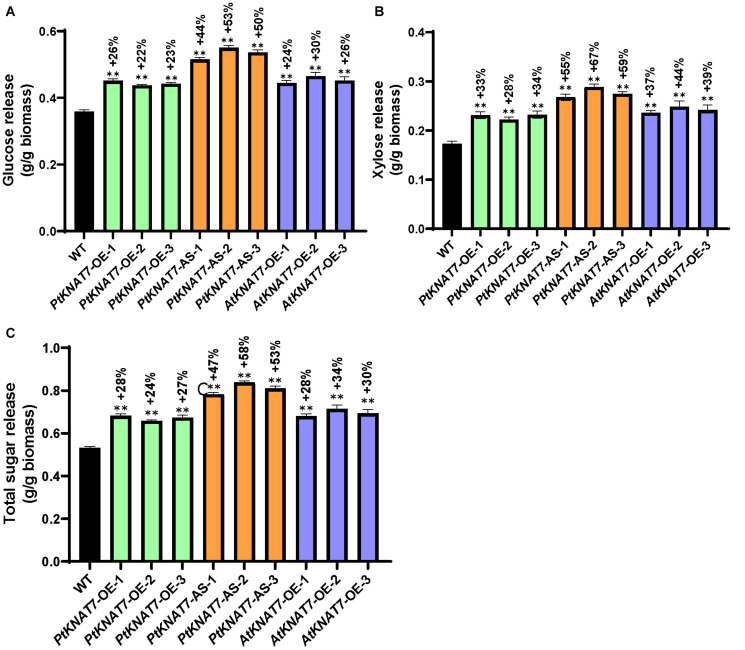
Saccharification yield of three PtKNAT*7*-OE, *PtKNAT7*-AS, and *AtKNAT7*-OE lines. **(A)** Glucose released, **(B)** xylose released, and **(C)** total sugars released from all the three independent *KNAT7* transgenic lines of poplars. Significant differences were observed when compared to WT controls, *P*-value < 0.01.

Saccharification efficiency measurements were performed to estimate simple sugar release from the wood samples obtained from greenhouse-grown transgenic and WT poplar plants. Glucose release was increased in all *KNAT7* transgenic lines as compared to WT controls. *PtKNAT7-OE* lines showed an overall 22–26% increase in glucose release whereas *PtKNAT7-AS* lines showed a 44–53% increase in glucose release over the WT controls (Figure 6A). There was also a 24–30% increase in glucose release for *AtKNAT7-OE* expression lines (Figure 6A). The data on xylose released also showed a similar trend of sugar release with a 28–34% increase in *PtKNAT7-OE* lines, 55–67% in *PtKNAT7-AS* lines, and 37–44% increase in *AtKNAT7-OE* lines (Figure 6B). The total increase in sugar release varied from 24 to 58% in these *KNAT7* transgenic lines and that could be mainly attributed to the alteration in the SCW formation that occurred as a result of changes in the expression of *KNAT7* genes (Figure 6C). Also, the increase in the S/G ratio and changes in lignin content might have contributed to the reduced cell wall recalcitrance.

## Discussion

Investigations into the role of KNAT7, one of the members of the plant KNOX II TF family, began with the *Arabidopsis* microarray data mining studies where the *KNAT7* gene was highly co-expressed with *Cellulose synthase A* (*CESA*) genes involved in the SCW formation (Brown et al., 2005; Ehlting et al., 2005; Persson et al., 2005). To understand the role of KNAT7 in SCW synthesis, Zhong et al. (2008) reported that dominant repression of AtKNAT7 TF exhibited reduced SCW thickening in interfascicular and xylary fibers and vessels (but no *irx* phenomenon) suggesting a positive regulation of SCW biosynthesis by KNAT7 TF. Overexpression of the *KNAT7* gene did not change the SCW thickness of vessels and fibers in transgenics compared to wild-type control plants (Zhong et al., 2008). Similarly, in another study by Pandey et al. (2016), overexpression of *NbKNAT7* in tobacco resulted in thickening of the SCW in the xylem while suppression of *NbKNAT7* in tobacco VIGS and *RNAi* plants resulted in thinner xylem SCWs coupled with increased xylem area. Furthermore, the positive regulation of xylan biosynthesis by KNAT7 TF has been recently reported in *Arabidopsis* inflorescence stems (He et al., 2018) and seed mucilage formation (Wang Y. et al., 2020). The use of constitutive promoters like CaMV 35S driving the expression of transgenes for such studies is known to produce several undesirable growth outcomes. In order to circumvent such growth phenotypes, Ko et al. (2012b) described the discovery of utility promoters, such as DX15 that drive strong developing xylem-specific expression of transgenes. A number of papers have described the use of DX15 promoters for biomass engineering (e.g., Cho et al., 2019). We have, therefore, used DX15-promoter for driving the expression of *KNAT7* transgenes in this work. In the present study, we overexpressed poplar and *Arabidopsis KNAT7* genes in transgenic poplars under the regulation of DX15 promoters and our observations suggest that KNAT7 acts like a positive regulator in poplars. It is also possible that there are species-specific differences in KNAT7 functions in SCW biosynthesis. Overall, the xylem area in *PtKNAT7-AS* lines increased by 26% while the xylem area was decreased in *PtKNAT7-OE* lines by 15% as compared to WT stems confirming the earlier observations in *NbKNAT7* transgenic lines of tobacco (Pandey et al., 2016), where the increased xylem proliferation in antisense lines could be partially attributed as a compensatory mechanism in response to reduced fiber cell wall thickness. However, in the present study, we did not observe any significant changes in the cell wall thickness in stems of *PtKNAT7* transgenic lines. It has not escaped our attention that *AtKNAT7-OE* transgenics showed xylem area phenotype similar to *PtKNAT7-AS* and not like *PtKNAT7-OE*. We are still far away from understanding the impact of such manipulations on plant phenotypes and it appears that at least, in this case, specific-specific variations in the *KNAT7* gene source might be responsible for this phenotype. But further investigations are warranted into this phenomenon in the future.

On the contrary, Li et al. (2012) initially claimed KNAT7 TF to be a negative regulator of SCW formation because *Atknat7* mutants had thicker interfascicular fibers but curiously also had *irx* phenotype in the vessels. However, these studies could not explain the *irx* phenotype in vessels when interfascicular fiber cells displayed SCW thickening at the same time. Recently, Qin et al. (2020) and Wang S. et al. (2020) elegantly reconciled these paradoxical observations by suggesting that KNAT3 and KNAT7 work synergistically in fibers but antagonistically in vessels in the regulation of SCW biosynthesis. As suggested by He et al. (2018), KNAT7 may not simply activate or repress every component of the secondary wall equally. Rather, it may differentially activate and suppress the deposition of various SCW components in specific cell types. Also, it is possible that KNAT7 functions either as an activator or repressor, depending upon the composition of the TF network active in different tissues or cell types of plants. Lastly, one cannot ignore species-specific variations in the transgenes that may result in different phenotypes.

We observed that overexpression of the *PtKNAT7* and *AtKNAT7* genes did not significantly increase the total lignin content although some monolignol biosynthesis genes, as well as some other representative genes involved in SCW formation, were upregulated. This observation is similar to Zhong et al. (2008) who also did not see significant changes in lignin quantities when the *AtKNAT7* gene was overexpressed. However, antisense *PtKNAT7* suppressed lines in poplars had lower lignin content and suppressed expression of SCW genes. This observation is again different than the earlier findings by Li et al. (2012), where they reported increased lignin deposition in Arabidopsis *knat*7 mutants. We also observed that S/G lignin ratios were significantly increased in both *PtKNAT7-OE, AtKNAT7-OE*, and *PtKNAT7-AS* lines. Considering the potential importance of KNAT7 TF in syringyl lignin biosynthesis (Qin et al., 2020; Wang S. et al., 2020), it is expected that overexpression of *KNAT7* may increase S lignin synthesis but it is puzzling why antisense suppression of *KNAT7* also increased S lignin content. It is possible that all the components of the TF network that interact with KNAT7 are not yet completely identified and there might be some complex regulation of S lignin synthesis or suppression of G lignin production that might be occurring in such transgenic lines.

Recently, Yoo et al. (2018) reported a negative correlation between lignin content and saccharification efficiency of poplar woody tissues and a positive correlation between S/G ratio and saccharification efficiency of SCW biomass. The current report is the first time when transgenic *KNAT7* poplar lines have been examined for their lignin quantities and content as well as saccharification efficiencies. We have observed increased saccharification efficiencies in all the transgenic lines of poplar altered in *KNAT7* gene expression compared to WT controls. The decrease in lignin content in *PtKNAT7-AS* lines and an increase in S/G ratios in all transgenic lines are positively associated with increased saccharification efficiencies. Similar to present findings, Pandey et al. (2016) also observed that tobacco *NbKNAT7* VIGS lines had increased saccharification efficiency than control plants.

While the focus of this study is on the biotechnological application of genetic manipulation of KNAT7, it is a common observation that in transgenic plants manipulated in lignin biosynthesis genes show abnormal growth phenotypes (e.g., Ko et al., 2012a). We observed that *KNAT7* transgenic poplar lines show almost similar or slightly better growth phenotypes compared to WT control plants. These observations are similar to an earlier report in tobacco *KNAT7* transgenic plants where normal-looking transgenic *KNAT7* plants were produced (Pandey et al., 2016). An increase in saccharification efficiencies, indicative of increased bioethanol production potential, without altering growth is a desirable characteristic of these *KNAT7* transgenic poplar lines from a bioenergy production perspective. In conclusion, the genetic alteration of *KNAT7* could be one of the powerful strategies for increasing saccharification efficiency in transgenic bioenergy plants and has a great potential for improving bioethanol production.

## Data Availability Statement

The original contributions presented in the study are included in the article/Supplementary Material, further inquiries can be directed to the corresponding author.

## Author Contributions

YA and CJ planned the experiments and interpreted the results. AN initiated the *KNAT7* experiments in poplars. AB performed saccharification assays. AH-W and CD did lignin analysis. All authors contributed to manuscript preparation.

## Author Disclaimer

The views expressed in the article do not necessarily represent the views of the DOE or the United States Government. The United States Government retains and the publisher, by accepting the article for publication, acknowledges that the United States Government retains a non-exclusive, paid-up, irrevocable, worldwide license to publish or reproduce the published form of this work or allow others to do so, for the United States Government purposes.

## Conflict of Interest

AN is currently employed by Kaveri Seeds. The remaining authors declare that the research was conducted in the absence of any commercial or financial relationships that could be construed as a potential conflict of interest.

## Publisher’s Note

All claims expressed in this article are solely those of the authors and do not necessarily represent those of their affiliated organizations, or those of the publisher, the editors and the reviewers. Any product that may be evaluated in this article, or claim that may be made by its manufacturer, is not guaranteed or endorsed by the publisher.
