# Supplementary material for: Genetic Modification of KNAT7 Transcription Factor Expression Enhances Saccharification and Reduces Recalcitrance of Woody Biomass in Poplars
Source: Front Plant Sci. 2021 Oct 26;12:762067. doi: 10.3389/fpls.2021.762067 (PMC8594486; doi:10.3389/fpls.2021.762067)
Supplement: Supplementary file 1 [file Table_1.DOCX]

**Supplementary Table 1: Primers used in this research are listed**

| **Primer Name** | **Primer Sequence** | |  |
| --- | --- | --- | --- |
| Pop-KNAT7-FP_XBaI | GTCTAGAATGCAAGAACCAAACTTGG | |  |
| Pop-KNAT7-RP_SacI | AGAGCTCCTACCTTTTGCGCTTG | |  |
| At-KNAT7-FP_XBaI | GTCTAGAATGCAAGAAGCGGCACTA | |  |
| At-KNAT7-RP_PacI | ATTAATTAAGTCCAAGCGCAAACACTAA | |  |
| Pop-KNAT7-FP_SacI | AGAGCTCATGCAAGAACCAAACTTG | |  |
| Pop-KNAT7-RP_XBaI | ATCTAGACTACCTTTTGCGCTTGGAC | |  |
| At-KNAT7-FP_PacI | CTTAATTAAATGCAAGAAGCGGCAC | |  |
| At-KNAT7-RP_XBaI | ATCTAGAGTCCAAGCGCAAACACTAA | |  |
| Pop-CCR-FP | GGGATATGGCTAAGGAGAAAGG | |  |
| Pop-CCR-RP | GAGGTACTTGAGGATGTGAGTG | |  |
| Pop-PAL-FP | GCTGGCCATGATGCTAGTAAT | |  |
| Pop-PAL-RP | GTTCCCTTGTCCATGCTATCC | |  |
| Pop-CesA8-FP | CGCAACAAACTCACACCATAC | |  |
| Pop-CesA8-RP | AGGCACTATCGACAGGATTTG | |  |
| Pop_IRX9_FP | GGTTATCGGCTGGCATCTAA | |  |
| Pop_IRX9_RP | GAGGTTTGCTGGACTGAAGA | |  |
|  | |  | |
|  | |  | |
