# Supplementary figures and images for: Genetic Modification of KNAT7 Transcription Factor Expression Enhances Saccharification and Reduces Recalcitrance of Woody Biomass in Poplars
Source: Front Plant Sci. 2021 Oct 26;12:762067. doi: 10.3389/fpls.2021.762067 (PMC8594486; doi:10.3389/fpls.2021.762067)

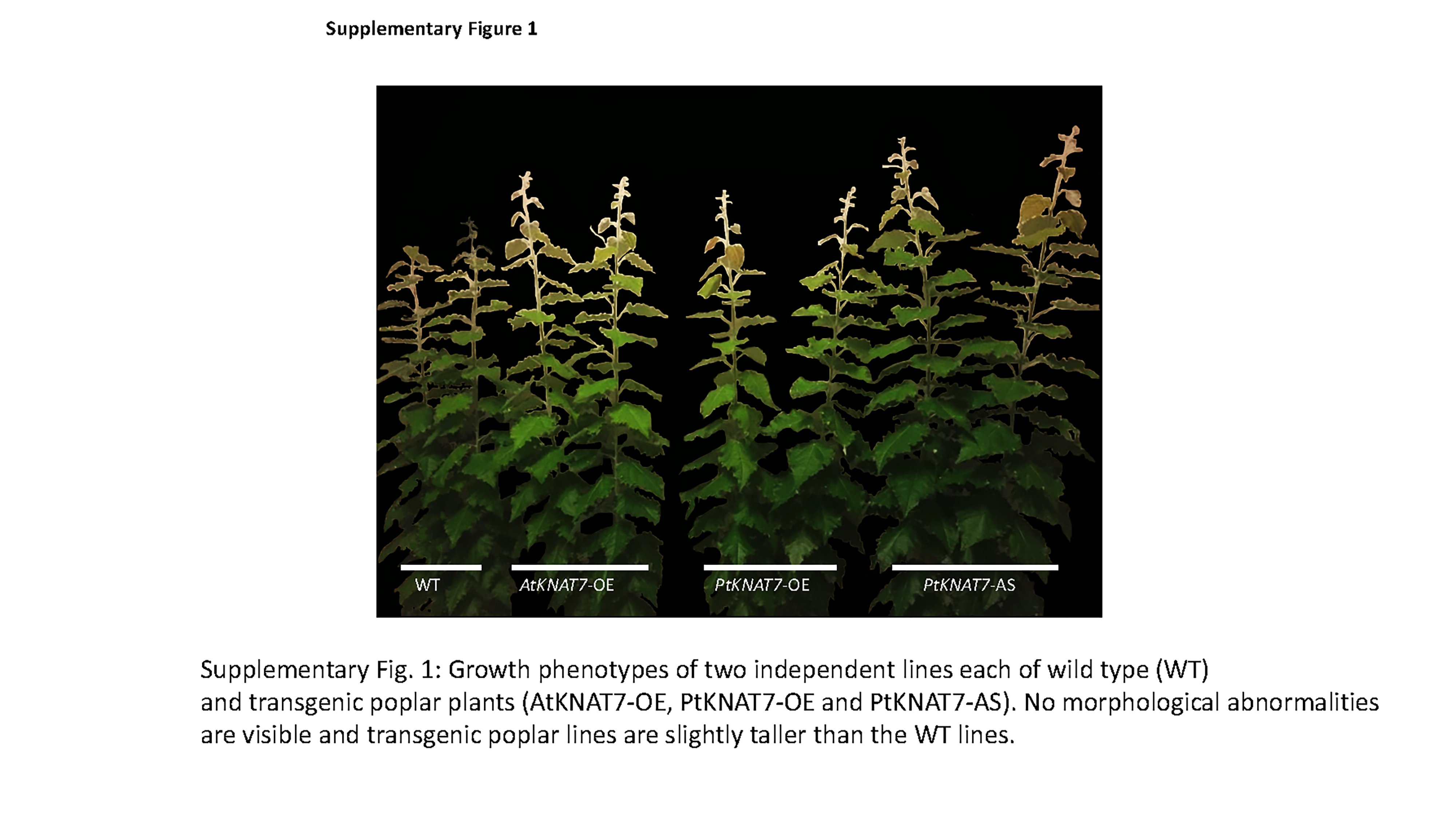

Supplement: Supplementary file 2 [file Image_1.tiff]
